# The Effect of Cataract on Eye Movement Perimetry

**DOI:** 10.1155/2015/425067

**Published:** 2015-05-20

**Authors:** G. Thepass, J. J. M. Pel, K. A. Vermeer, O. Creten, S. R. Bryan, H. G. Lemij, J. van der Steen

**Affiliations:** ^1^Vestibular and Ocular Motor Research Group, Department of Neuroscience, Erasmus MC, P.O. Box 2040, 3000 CA Rotterdam, Netherlands; ^2^Rotterdam Ophthalmic Institute, P.O. Box 70030, 3000 LM Rotterdam, Netherlands; ^3^Rotterdam Eye Hospital, P.O. Box 70030, 3000 LM Rotterdam, Netherlands; ^4^Department of Biostatistics, Erasmus MC, P.O. Box 2040, 3000 CA Rotterdam, Netherlands; ^5^Royal Dutch Visio, P.O. Box 1180, 1270 BD Huizen, Netherlands

## Abstract

*Purpose*. To determine how different grades of cataract affect sensitivity threshold and saccadic reaction time (SRT) in eye movement perimetry (EMP). *Methods*. In EMP, the visual field is tested by assessing the saccades that a subject makes towards peripheral stimuli using an eye tracker. Forty-eight cataract patients underwent pre- and postoperative EMP examination in both eyes. The subjects had to fix a central stimulus presented on the eye tracker monitor and to look at any detected peripheral stimulus upon its appearance. A multilevel mixed model was used to determine the factors that affected the sensitivity threshold and the SRT as a function of cataract grade. *Results*. We found no effect of cataract severity (LOCS III grades I through IV) on SRT and the sensitivity thresholds. In cataract of LOCS III grade V, however, we found an increase by 27% and 21% (*p* < 0.001), respectively, compared to the SRT and the sensitivity threshold in LOCS III grade I. Eyes that underwent cataract surgery showed no change in mean SRTs and sensitivity thresholds after surgery in LOCS III grade IV and lower. *Conclusion*. The present study shows that EMP can be readily used in patients with cataract with LOCS III grade IV and below.

## 1. Introduction

Standard automated perimetry (SAP) has become the standard of care in assessing visual fields. Both clinicians and patients agree that better and less demanding tests are desirable [1]. We recently developed an eye movement perimetry (EMP) test that uses an eye tracking system [2]. EMP takes advantage of the natural reflex to direct our gaze with a saccadic eye movement towards a visual stimulus that appears in our peripheral visual field [3, 4]. Such saccadic responses are relatively uncontaminated by the subject's uncertainty resulting from task instructions, as saccades are in practice performed with little awareness by the subject. Monitoring of this saccade with an eye tracker offers the objective registration of stimulus detection and the possibility to determine additional parameters. One such parameter is the saccadic reaction time (SRT) [5]. The SRT depends on many factors, such as stimulus intensity, diameter, and eccentricity [2, 6–8]. SRTs in visual field testing show little variability at various locations in a 30-degree visual field [2, 9]. In glaucoma, a disorder that progressively affects the retinal ganglion cells and their axons, an increasingly larger area of the visual field becomes irreversibly damaged [10]. This change in visual field integrity is most commonly tested with SAP, which tests retinal sensitivity thresholds in predefined locations. EMP provides a quantitative measure of visual field responsiveness and may therefore contribute to the early detection of glaucomatous damage in parts of the retina [11]. Relatively little is known about any confounding factors in EMP. A likely confounding factor is cataract, a condition that is highly prevalent in the elderly population in which also glaucoma is common [12, 13]. If cataract was indeed a significant confounder in EMP, it would limit the clinical use of EMP. All cataract subtypes affect contrast sensitivity and visual acuity (VA) [13, 14] and cataract affects the main outcomes of SAP [15–20].

EMP has the potential to become an acceptable visual field test to patients. The objective of this study was to obtain limits of its applicability in patients with cataract. To reach this objective, we performed EMP in subjects with various grades of cataract severity (grades I to VI of the Lens Opacity Classification System III (LOCS III) grading) [21], before and after cataract surgery.

## 2. Methods

### 2.1. Subjects

The subjects were selected from patients eligible for cataract extraction by phacoemulsification at the Rotterdam Eye Hospital, Netherlands. They had to be at least 40 years old. Unilateral pseudophakic patients or patients with a history of ocular or systemic disorders (other than cataract) with known effects on visual acuity or visual field were excluded. There were no restrictions on the degree of cataract. The experimental procedures were approved by the Medical Ethics Committee of the Erasmus University Medical Centre, Rotterdam, Netherlands [MEC-2009-199] as well as by the institutional review board at the Rotterdam Eye Hospital, Rotterdam, Netherlands. The study adhered to the Declaration of Helsinki for research involving human subjects. Subjects provided written informed consent.

After routine preoperative ophthalmic and systemic examination, all subjects underwent monocular EMP testing of each eye before the cataract surgery. The best-corrected visual acuity (LogMAR) was measured before the test by using the Early Treatment Diabetic Retinopathy Study (ETDRS) system. The grading of the cataract was done by any of three ophthalmologists specialized in cataract surgery at the Rotterdam Eye Hospital, based on the LOCS III classification system [21]. Each subject was allocated to one of six LOCS III grades based on the single highest score, on a scale from I to VI, of each of the subtypes: nuclear opacification, nuclear color, cortical cataract, or subcapsular cataract. In 47 eyes, a standard monofocal lens (SA60AT, Alcon Laboratories, Inc., Fort Worth, USA) and in one eye an ultraviolet and blue light blocking monofocal lens (SN60WF, Alcon Laboratories, Inc., Fort Worth, USA) were implanted. The eyes that had undergone cataract surgery were assumed to have no remaining lens opacities. The forty-eight subjects successfully underwent uncomplicated cataract surgery. After a mean recovery period of 33.4 days (SD 9.1) following the surgery, the EMP testing was repeated.

### 2.2. EMP

The eye tracking and stimulus presentation system that we used was the Tobii T60-XL (Tobii Technology AB, Danderyd, Sweden). The device consisted of a liquid crystal display (LCD) that used integrated near-infrared cameras to follow the subject's gaze on the display. The sample rate of the device was 60 Hz. A stimulus sequence was made in which the stimuli were presented in a random order. This standard sequence was used in all exams. The sequence was shown with Tobii Studio software running on a laptop computer (Dell M6400). The latency of the eye tracking system was 33 ms (Tobii T60 XL Eye Tracker manual, revision 2). The LCD was placed at a fixed distance of 55 cm from the subjects. The individual refraction (spherical equivalent) was corrected by using a single correction glass. EMP was performed under monocular viewing conditions by covering one eye with a black polymethyl methacrylate (PMMA) plate that blocked the vision of that eye, but allowed the eye tracker to use both eyes for gaze recording. Afterwards, the PMMA plate was shifted to the other eye, and the exam was repeated. All sessions took place in the same room, the background luminance was kept constant with normal room lights switched off, and noise levels were kept to a minimum to avoid distraction. The standardized pretest instructions given to the subjects were as follows: first fixate the stimulus at the center of the screen; when a peripheral stimulus appears, look towards it and continue to look at that stimulus until it disappears and then fixate the central stimulus again.

A standard 5-point eye movement calibration procedure was run prior to each exam. Next, a short practice run was carried out so that the subjects could familiarize themselves with the test. Thereafter, an additional 5-point calibration was carried out for data processing. This second calibration was used to align the stimuli sequence presented on the screen with the gaze coordinates of the eye tracker, as in earlier experiments misalignment of the gaze coordinates sometimes occurred.

**Figure 1:**
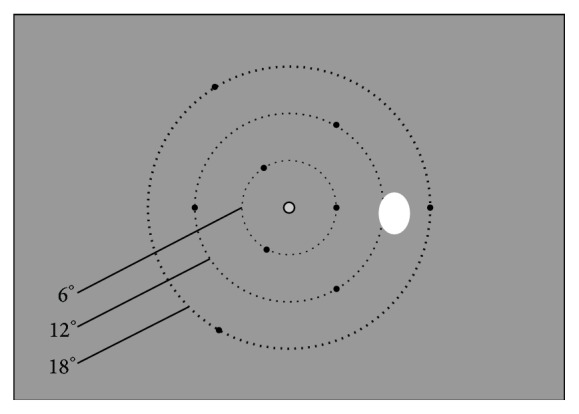
Stimulus grid showing the nine test locations (black dots) on the three concentric grid lines at 6°, 12°, and 18° (not visible in actual test), the central fixation point, and the reference location of the blind spot (white oval).

During each exam, 108 unique stimuli were presented on the screen by using an overlap paradigm, where the central stimulus remained lit when the peripheral stimulus appeared. The peripheral stimuli were presented for 1.2 seconds on the monitor at 4 different luminance levels (70, 80, 90, and 100% on a grey to white scale, corresponding to 210, 300, 385, and 475 cd/m^2^, resp.) and 3 different diameters (0.34°, 0.58°, and 1.15° of visual angle similar to standard Goldmann size III, IV, and V stimuli, resp.). The background was kept at a constant luminance of 60% (160 cd/m^2^). Because we used a flat monitor, the peripheral stimulus diameter on the screen was also corrected for eccentricity. The stimulus grid consisted of nine locations, three on each of the concentric circular gridlines at 6°, 12°, and 18° (see Figure 1). The test grid that was used was specifically designed to test the effect of cataract on EMP and not to test the visual field. The number of test locations was limited to nine locations to enable the repetition of stimuli of the same intensity and diameter at a specific location, while keeping the duration of the exam limited. For example, testing all locations of the commonly used Humphrey Field Analyzer 24-2 grid (Carl Zeiss Meditec AG) would have taken over 60 minutes to complete. All the 108 stimuli in the sequence were shown twice in each exam and the order and the time between stimulus presentations were randomized. The total duration per exam, consisting of 216 trials, was approximately 11 minutes.

### 2.3. Data Analysis

A previously published Matlab (MathWorks, Natick, MA, USA) decision algorithm was used for automated, offline processing [2]. The algorithm compared gaze coordinates to the coordinates of the presented peripheral stimuli. For all trials, the gaze path from the central stimulus to the peripheral stimulus was visually checked. If the saccadic eye movement did not start on the central stimulus, it did not end at the peripheral stimulus or when no eye movement data was available due to poor eye tracking, the trial was excluded from the analysis. To correct for eye tracking inaccuracy and any over- and undershoot of the primary saccade, the saccadic eye movement was included when, at onset, it was in the stimulus direction and covered >50% of the total central to peripheral stimulus distance. Trials therefore were either excluded by the investigator due to incorrect eye movements (searching for stimuli) or on the basis of missing data samples due to eye tracking failure.

**Figure 2:**
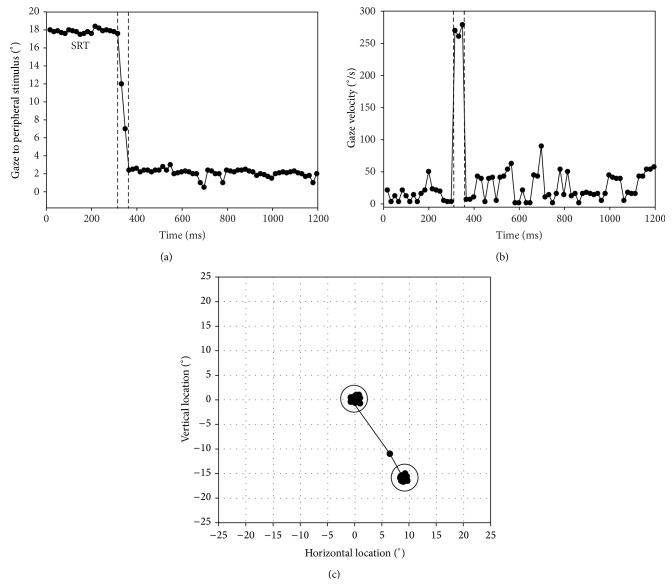
Offline processing interface showing the gaze path of a single trial. Panel (a) shows the gaze location of the subject. At ~360 ms an accurate saccade is made towards the stimulus. Panel (b) visualizes the eye velocity and shows the saccade and the velocity based saccade onset and end (vertical lines). Panel (c) shows the gaze coordinates on the test grid. The central circle represents the central fixation point area and the peripheral circle the stimulus area. The line depicts the gaze of the subject.

For each detected stimulus, the SRT was calculated as the difference between the moment of stimulus presentation and saccadic onset, which was set at an eye velocity of >50° per second. Figures 2(a), 2(b), and 2(c) show the typical gaze path towards a stimulus that was correctly labeled as “seen.” The SRT value of the repeated stimulus in each exam was only used if the data of the first stimulus was missing (e.g., due to eye tracking failure or searching for stimuli). When neither of the trials led to the registration of a SRT, the outcome was treated as missing data. The sensitivity thresholds were determined from the lowest seen stimulus intensity for each diameter on each tested location. Whenever a subject blinked or the eye tracker lost signal, some of the data samples were lost. This loss was quantified as a signal quality parameter, which was the percentage of recorded data samples as a part of the total number of eye tracker data samples.

### 2.4. Statistics

Because of the hierarchical data structure (described below), a multilevel mixed model was used (generalized linear mixed model, SPSS, IBM) to determine the influence of the factors on the dependent variables: sensitivity threshold and SRT. This linear regression model took both the within subject and between subject variability into account by allowing a leveled structure. Three levels were used: (1) the subject (48 subjects), (2) the eye (two eyes per subject), and (3) the stimulus location (9 locations per eye). The following individual factors were included in the model: the age of the subject and the eye tracker signal quality as continuous variables and gender, session, stimulus intensity, stimulus diameter, stimulus eccentricity, and LOCS III grade as categorical variables. LogMAR was not added as a factor to the model because of its strong correlation with the LOCS III grade. Any differences between the levels within individual factors were tested with pairwise contrast estimates. This model was applied to both dependent variables. For all experiments, a 5% significance level was used.

## 3. Results

### 3.1. Eye Tracking

Sixty-nine subjects were selected for inclusion in the current study. Twenty-one of these were excluded from the analysis (see Table 1 for details). Subject characteristics of the remaining 48 subjects have been shown in Tables 2 and 3. In 3 exams (2 subjects) the eye tracker was unable to collect gaze data. These 3 exams were excluded from the analysis, and their data was treated as missing data. The median eye tracker signal quality of the remaining 48 subjects was 93% (95% confidence interval (CI) 91%–94%) (see Table 4). The signal quality was not correlated with the LOCS III grade (Spearman *ρ* = −0.038, *p* = 0.61). The eye tracking signal quality in the eyes after cataract surgery (93% (95% CI: 90%–95%)) was not statistically significantly different from preoperative values.

### 3.2. Saccadic Reaction Times

**Figure 3:**
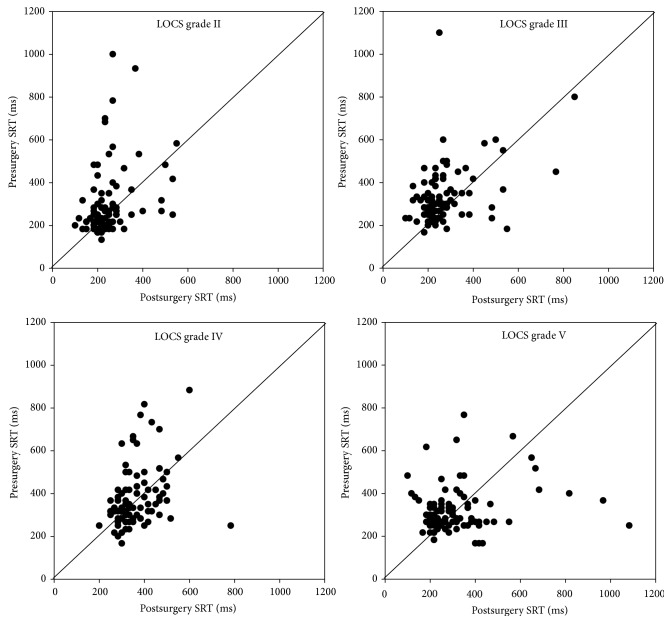
Scatterplots showing pre- versus postsurgery SRT values (ms) assessed in the same eye (108 stimuli) of four patients with different cataract severity (LOCS III grades II through V).

**Figure 4:**
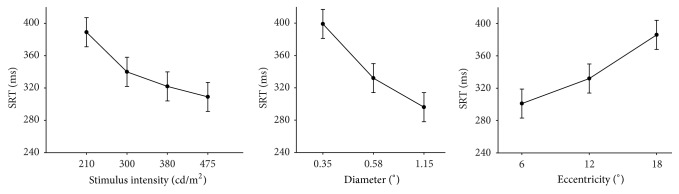
Estimated mean SRTs and their corresponding 95% confidence intervals for stimulus intensity, stimulus diameter, and stimulus eccentricity. (Other factors were fixed at 69 years, 91% signal quality and first session.) Pairwise contrasts showed statistically significant differences between all levels of each factor.

**Figure 5:**
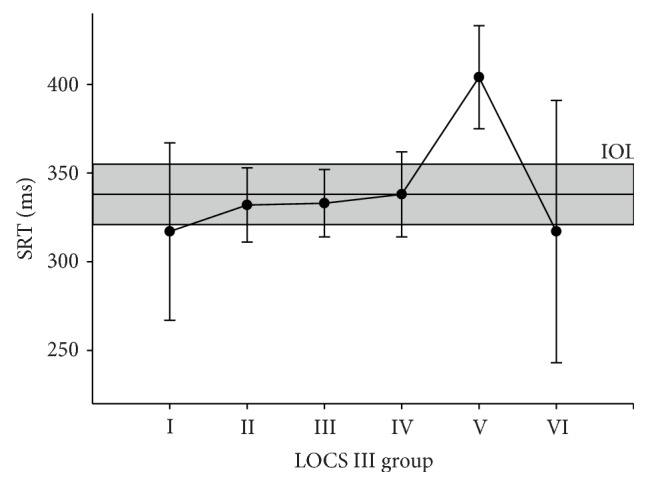
Estimated mean SRTs and their corresponding 95% confidence intervals for the 6 LOCS III grades. The estimated postoperative mean SRTs of the pseudophakic eyes (IOL) have been depicted by the continuous band with its corresponding 95% confidence intervals. Pairwise contrasts showed statistically significant differences between LOCS III grade V and all other grades (*p* < 0.05).

For all detected stimuli, the SRTs were determined and evaluated in a multilevel model.  Figure 3 shows the inter- and intrasubject variability of SRTs before and after cataract surgery in four patients with different cataract severities. Estimated mean SRTs with corresponding 95% confidence intervals have been presented in Figures 4 and 5. The figures show the relationship between the SRT and stimulus intensity, stimulus diameter, stimulus eccentricity, and LOCS III grade. The SRT went up with dimmer stimuli (*p* < 0.001), smaller stimuli (*p* < 0.001), and a larger stimulus eccentricity (*p* < 0.001). We found longer SRTs in LOCS III grade V (*p* < 0.001) but not in the other LOCS III grades (*p* > 0.05). In the second session, SRTs were shorter than in the first session (*p* < 0.001). Age, gender, and eye tracking signal quality did not statistically significantly correlate with SRT. For a comprehensive overview of the model coefficients for all factors and their levels, see Table 5.

The differences between the within-factor levels were tested by pairwise contrast estimates as a post hoc test in the generalized linear mixed model. These tests showed statistically significant differences (*p* < 0.001) between all levels of the factors stimulus intensity, stimulus diameter, and stimulus eccentricity. The estimated mean SRTs for the 6 LOCS III grades are shown in Figure 5. SRTs increased with higher LOCS III grade compared to grade I by 15 ms, 16 ms, 21 ms, 87 ms, and 0 ms for grades II through VI, respectively. By pairwise contrast tests, differences were tested between the six levels of the LOCS III grade. Grade V was the only grade that showed statistically significantly longer SRTs compared to the other LOCS III grades (*p* < 0.05). The pseudophakic eyes (grey band in Figure 5) showed SRTs similar to those found in all the LOCS III grades, except for grade V (*p* < 0.05).

### 3.3. Sensitivity Threshold

**Figure 6:**
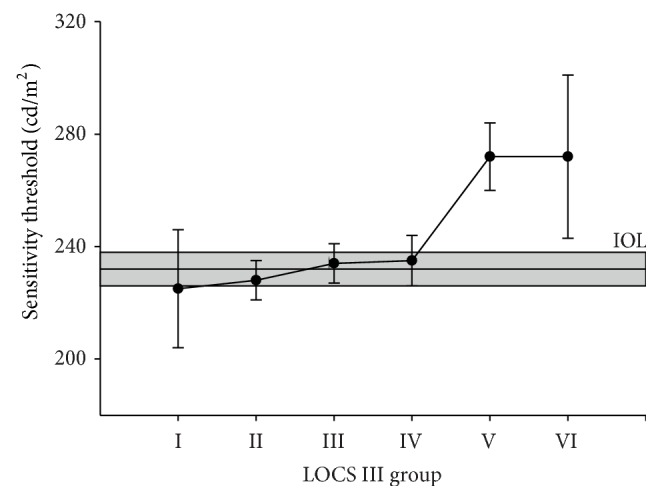
Estimated mean sensitivity (cd/m^2^) and their corresponding 95% confidence intervals for the 6 LOCS III grades. The estimated postoperative mean sensitivity of the pseudophakic eyes has been depicted by the continuous band with its corresponding 95% confidence intervals. Pairwise contrasts showed statistically significant differences between LOCS III grades V (*p* < 0.001) and VI (*p* < 0.05) and all other grades.

The sensitivity thresholds increased with the LOCS III grade (*p* < 0.001). This increase was most notable for grades V and VI (Figure 6; *p* < 0.001; Table 5). The sensitivity thresholds went up with smaller stimulus diameters, with larger stimulus eccentricity, and with a lower eye tracking signal quality (*p* < 0.001 for all 3 factors). The sensitivity threshold was lower in the second session (*p* < 0.001). Compared to LOCS III grade I, the threshold increased by 3 cd/m^2^, 9 cd/m^2^, 10 cd/m^2^, 47 cd/m^2^ and, 47 cd/m^2^, respectively, for grades II through VI. The sensitivity thresholds were only statistically significantly raised in eyes with LOCS III grades of V (*p* < 0.001) or VI (*p* < 0.05) compared to each of the other 4 levels. Figure 6 shows postoperative results similarly displayed as for the SRT in Figure 5; eyes with lower grade cataract did not have any statistically significantly higher sensitivity thresholds compared to eyes with an implanted IOL. The increase in sensitivity threshold was statistically significant in LOCS III grades V (*p* < 0.001) and VI (*p* < 0.005).

## 4. Discussion

### 4.1. Effect of Cataract on SRT

While SAP has been the standard of care in assessing visual fields for several decades, physicians and patients share the desire for more reliable and patient-friendly tests [1]. New technology, such as eye tracking, offers alternative means to perform visual field testing. This also allows SRT registration which has shown to potentially improve speed and accuracy in standard perimetry [5]. Because cataract often coincides with other eye conditions such as glaucoma [12], our aim was to quantify the possible confounding effect of cataract in eye movement perimetry measurements. Only advanced cataract was shown to significantly increase the SRT (LOCS III grade V) and the sensitivity thresholds (LOCS III grades V and VI) by approximately 25%. In all other cases, cataract did not significantly confound EMP in our data set.

### 4.2. SRT

The effects of stimulus properties on SRT have been studied extensively in eye movement studies in the past. Higher stimulus intensity shortens SRT [2, 8, 22–24]. Several studies concluded that a larger stimulus shortens SRT [2, 22], although others have not found such an effect [25]. Likewise, some studies have shown that SRTs increase with eccentricity [2, 26, 27], although other studies have reported that SRTs are independent of eccentricity [24, 25]. In a previous study, we have also found that brighter stimuli result in shorter SRTs and that more eccentric stimuli yield longer SRTs [2]. These contradictory results in the literature suggest that stimulus properties therefore may have to be taken into account when interpreting SRTs in EMP.

Aside from intensity and eccentricity, SRTs in EMP can also be affected by severe cataract. Our results showed that cataract LOCS III grade V led to statistically significantly longer SRTs. Although the visual stimuli obviously become increasingly difficult to detect with cataract-induced loss of contrast [28], no correlation was found between lower grades of cataract and SRT. Only for LOCS III grade V there was a sudden increase in SRT. This finding is in line with SRT prediction models which show that contrast has little effect on SRT until the contrast threshold is reached [29, 30]. Each subject was allocated to one of six LOCS III grades based on the single highest score, on a scale from I to VI, of each of the subtypes: nuclear color, cortical cataract, or subcapsular cataract. Lam et al. found that subcapsular cataract did correlate and nuclear or cortical cataract did not significantly correlate with the recovery of the visual field after cataract surgery [16]. The optical effects of subcapsular cataract are therefore substantially different from those of nuclear and cortical cataract. In our LOCS III grade V patients, we indeed found a higher occurrence of posterior subcapsular cataract, which may explain why the SRTs stood out in this particular grade.

### 4.3. Sensitivity Threshold

Previous studies have shown that cataract causes a general increase in sensitivity threshold in SAP [16, 19]. It has also been shown that cataract can produce relative visual field defects that hide (glaucomatous) visual field damage and progression [18, 20]. In SAP, the fairly spatially uniform [16] reduction of sensitivity can be resolved by cataract surgery that improves the mean sensitivity threshold [17–20, 31]. Based on the similarities between SAP and EMP, we expected that in our study cataract would cause a gradual increase in sensitivity threshold in EMP. However, only small and statistically insignificant increases in sensitivity threshold were found in LOCS III grades I through IV. Surprisingly, the sensitivity threshold increases in LOCS III grades V and VI were much larger. This means that, in LOCS III grades V and VI, the dimmer stimuli (mainly 210 cd/m^2^) become notably more difficult to detect, which leads to an increase in the sensitivity threshold.

Despite several obvious similarities between EMP and SAP, there are clear differences between the two techniques. The most prominent difference is that subjects in EMP respond to peripheral stimuli with an eye movement instead of pressing a button. Using reflexive responses probably leads to increased test reliability. Secondly, the range of stimulus intensities that can be presented by both systems is different. The maximum luminance of the LCD that we used was 475 cd/m^2^, which is modest compared to the range of the Humphrey Field Analyzer which covers approximately 0.03 cd/m^2^ to 3200 cd/m^2^. However, within the limited luminance range that we used, we were able to detect differences in SRT and also in sensitivity thresholds. This suggests that the luminance range that we used may be of use in clinical or otherwise psychophysical testing of the oculomotor system. It is likely that the effects of cataract on SRT largely depend on the stimulus luminance. However, it is unclear if a wider luminance range would be of additional value in this experiment. We believe that other differences between SAP and EMP, such as the different lighting techniques (LCD monitor in EMP and surface illumination in SAP) and a difference in the distance between the subject and the screen, are unlikely to be a source for differences in the outcome of the test, as long as the perceived light intensities and the angular amplitudes between the stimuli are identical. An additional benefit of EMP is that the reproducibility of measurements in the periphery of the visual field has been proven to be better than in SAP [2].

### 4.4. Eye Tracking in Patients with Cataract

In EMP, it is critically important that the eyes are sufficiently tracked by the eye tracker [2]. A lower percentage of recorded data samples did not affect the SRT but it did show a correlation with a lower sensitivity threshold. The effect of cataract on the near-infrared eye tracking has not been reported in the literature before. In the present study, we found high tracking percentages of approximately 93% in all LOCS III grades and pseudophakic eyes, indicating that there is no significant effect of cataract and cataract surgery on the performance of the eye tracking system.

### 4.5. Study Limitations

The forty-eight subjects that were included in this study were not evenly distributed over the 6 LOCS III grades, because notably the higher grades are relatively rare in a western population. Although we did not find a significant statistical correlation for LOCS III grades I and VI in SRT and grade I in the sensitivity threshold, it should be taken into consideration that there are a small number of eyes evaluated in LOCS III grades I and VI (three and two eyes, resp.). Therefore, future studies on this topic should aim to include greater numbers of subjects with low and high grades of cataract, in order to determine if statistical significant correlations can be demonstrated in these grades.

The mixed model allowed us to evaluate the SRT and sensitivity threshold differences between the LOCS III grades in addition to the difference before and after cataract surgery for both the treated and the fellow eyes. In the fellow eyes, we found a small effect of cataract surgery on the SRT. We believe that a very small learning effect may have occurred. In the first session, the patients were possibly more anxious since they were tested in the week before surgery, while in the second session they were more familiar with the study procedures. A learning effect also occurs in SAP where patient experience may lead to an apparent improvement of the visual field [32–34]. Although previous work has not shown a learning effect in EMP [2], we cannot rule out its presence in this experiment.

## 5. Conclusions

In this paper, we have evaluated cataract as a probable and very common confounder in EMP measurements and we have shown that EMP can be used in all 6 cataract LOCS III grades. No effect of cataract was found for mild and moderate cataract. In LOCS III grades V and VI, however, EMP results should be interpreted more cautiously due to the confounding effect of cataract. In EMP, the SRT provides alternative visual field information to the standard outcome of visual field testing.

## Figures and Tables

**Table 1:** The number of subjects in the study and the reasons for exclusion of subjects.

Subjects selected for inclusion	*n* = 69
Not included in analysis	*n* = 21
Missing data	*n* = 1
Technical failure	*n* = 4
Other eye pseudophakic	*n* = 2
Refrain from participation	*n* = 9
Glaucoma	*n* = 3
Loss to follow-up	*n* = 2

**Table 2:** Demographics of the study population.

Women (*n*, %)	26 (54%)
Treated eye OD (*n*, %)	25 (52%)
Mean pre-op interval in days (SD)	4.9 ± 9.9
Mean post-op interval in days (SD)	33.4 ± 9.1
Mean LOCS III grade for treated eyes (SD)	3.5 ± 1.1
Mean LOCS III grade for fellow eyes (SD)	3.0 ± 1.1

OD: oculus dexter, right eye, LOCS III: Lens Opacity Classification System II.

**Table 3:** Number of eyes and the mean subject age stratified per LOCS III grade.

		Mean	LOCS1	LOCS2	LOCS3	LOCS4	LOCS5	LOCS6
Treated	Eyes (*n*)	8	1	9	17	10	10	1
	Age years (SD)	69.4 ± 7.7	55.1	70.4 ± 6.4	69.7 ± 7.3	69.8 ± 4.7	66.9 ± 9.0	82.6

Fellow	Eyes (*n*)	8	2	16	16	9	4	1
	Age years (SD)	69.4 ± 7.7	69.9 ± 3.7	67.4 ± 8.6	70.6 ± 6.6	67.7 ± 6.9	74.3 ± 7.2	82.6

LOCS III: Lens Opacity Classification System III.

**Table 4:** The signal quality of the eye tracker device stratified per LOCS III grade.

	Median	LOCS1	LOCS2	LOCS3	LOCS4	LOCS5	LOCS6	IOL
Signal quality % [CI_95%_]	93 [91–94]	96 [95–98]	91 [88–94]	93 [91–95]	92 [86–94]	93 [84–97]	98 [70–99]	93 [90–95]

LOCS III: Lens Opacity Classification System III.

**Table 5:** The results of the multilevel model of the individual factors (bold) and their levels.

	Saccadic reaction time (ms)			Sensitivity threshold (cd/m^2^)		
	Parameter estimate	95% CI	*p* value	Parameter estimate	95% CI	*p* value
*Intercept *	350	197 to 504	**<0.001**	215	178 to 252	**<0.001**
Age	−0	−2 to 2	**0.986**	0.04	−0.49 to 0.57	**0.893**
Signal quality	−1	−1 to 0	**0.233**	1.12	0.78 to 1.46	**<0.001**
Gender			**0.536**			**0.914**
Female	−9	−37 to 19	0.536	0.4	−7.6 to 8.5	0.914
Male	Reference			Reference		
Session			**<0.001**			**<0.001**
First	Reference			Reference		
Second	−30	−48 to −13	< 0.001	−8.3	−15.2 to −1.4	<0.05
Stimulus intensity			**<0.001**	(Not a factor in this model)		
70%	80	75 to 84	<0.001			
80%	31	26 to 35	<0.001			
90%	13	8 to 17	<0.001			
100%	Reference					
Stimulus diameter			**<0.001**			**<0.001**
0.35°	95	88 to 102	<0.001	34.1	31.1 to 37.1	<0.001
0.58°	36	30 to 41	<0.001	7.3	4.4 to 10.3	<0.001
1.15°	Reference			Reference		
Stimulus eccentricity			**<0.001**			**<0.001**
6°	−85	−93 to −78	<0.001	−23.3	−27.0 to −19.7	<0.001
12°	−54	−61 to −46	<0.001	−21.1	−24.7 to −17.4	<0.001
18°	Reference			Reference		
LOCS III grade			**<0.001**			**<0.001**
1	−20	−71 to 30	0.423	−7.3	−28.7 to 14.1	0.505
2	−6	−26 to 14	0.555	−4.7	−13.4 to 4.0	0.293
3	−5	−23 to 13	0.592	1.9	−6.4 to 10.1	0.659
4	0	−23 to 24	0.992	2.8	−7.4 to 13.1	0.587
5	66	37 to 95	<0.001	39.3	26.2 to 52.4	<0.001
6	−20	−94 to 53	0.587	39.3	10.2 to 68.4	<0.05
IOL	Reference			Reference		

LOCS III: Lens Opacity Classification System III and CI: confidence interval.

## Abbreviations

EMP: Eye movement perimetry
SRT: Saccadic reaction time.
